# Optimization of MCM-41 Mesoporous Material Mixed Matrix Polyethersulfone Membrane for Dye Removal

**DOI:** 10.3390/membranes11060414

**Published:** 2021-05-30

**Authors:** Rana J. Kadhim, Faris H. Al-Ani, Qusay F. Alsalhy, Alberto Figoli

**Affiliations:** 1 Civil Engineering Department, University of Technology-Iraq, Alsinaa Street 52, Baghdad 10066, Iraq; rana1979.kadhim@gmail.com (R.J.K.); 40027@uotechnology.edu.iq (F.H.A.-A.); 2 Membrane Technology Research Unit, Chemical Engineering Department, University of Technology, Alsinaa Street 52, Baghdad 10066, Iraq; 3 Institute on Membrane Technology, National Research Council (ITM-CNR), 87030 Rende, Italy; a.figoli@itm.cnr.it

**Keywords:** polyethersulfone, MCM-41 mesoporous, optimization, dyes, nanofiltration

## Abstract

The aim of this work is the optimization of the operating conditions under which MCM-41-mesoporous material can be incorporated into polyethersulfone (PES)/MCM-41 membranes for nanofiltration (NF) applications. MCM-41 mesoporous material mixed matrix PES membranes have the potential to reduce membrane fouling by organic dye molecules. Process optimization and modeling aim to reduce wasted energy while maintaining high flow during the operation to handle the energy efficiency problems membranes often have. An optimization technique was applied to obtain optimum values for some key parameters in the process to produce a certain amount of flux above the desired values. Response surface methodology (RSM) and analysis of variance (ANOVA) were used as mathematical and statistical analyses to improve the performance of the process on a larger scale. This work investigated the influence of the operating parameters, such as the feed pH values (3–11), MCM-41 content (0–1 wt.%), and the feed dye concentration (10–100 ppm) for each of the two studied dyes, acid black 210 (AB-210) and rose bengal (RB), and their interactions on the PES membrane permeability. The results showed that the PES membrane had the best performance at 64.25 (L·m^−2^·h^−1^·bar^-1^) and 63.16 (L·m^−2^·h^−1^·bar^-1^) for the AB-210 and RB dyes, respectively. An MCM-41 content of nearly 0.8 wt.% in the casting solution, feed dye concentration of 10 ppm for the studied dyes, and feed pH of 3 for the RB dye was found to be the optimal parameters for eliciting the response. The pH had no significant influence on the response for the AB-210 dye, while the pH shows some minor effects on response with the RB dye, and the Pareto chart of the standardized effects on the permeation flux of both dyes using statistically significant at the 5% significance level support these results.

## 1. Introduction

In general, the environment and the health of living organisms have been threatened by the release of large amounts of organic products (e.g., dyes) into wastewater by the leather industry at concentrations 10–150 ppm. As this effluent commonly pollutes water and is highly toxic, it is critical to remove dyes using modern and maximally effective techniques [1,2].

Environmentally, acidic (i.e., anionic) dyes belong to a highly problematic class of dyes. Acid black 210 (C_34_H_25_Na_2_N_11_O_11_S_3_) and rose bengal (C_20_H_2_Cl_4_l_4_Na_2_O_5_) are examples of anionic dyes that are widely used to alter the color of a solution, and extensive physical, chemical, and biological methodologies have been suggested to remove such dyes from wastewater [3,4,5].

Membrane technology has been employed for the removal of dyes and has been applied in water purification, wastewater recycling, and food industries, etc. [6]. It has good removal potential, and the materials can be reused. The main benefits of using membrane technology are its low operating cost, lower energy usage, a small area required for use, need for fewer chemical additives to remove impurities, improved production efficiency, and quality control [7,8].

Recently, many mesoporous materials have been used in nanocomposite membranes. The primary mesoporous material (MCM-41 as it is commonly called) is made of silica, due to its unique properties, including physicochemical properties, such as having a unique pore size, better structural flexibility, and excellent hard crystals [9,10]. Separation of organic solutes by NF membranes is conducted based on some mechanisms such as size exclusion, electrostatic charge repulsion, and adsorption phenomena on the membrane surface. Electrostatic charge interactions between charged solutes and charges on the membrane surface have the most important influence on the retention of the charged solutes compared to the size exclusion. These interactions cause a higher rejection of small charged solutes compared to what might be expected based on the size exclusion effects. On the other side, the adsorption of hydrophobic organic compounds is significant in the filtration of ionizable compounds when are electrostatically neutral. These beneficial properties are due to the effective silanol groups and the high specific surface of silica particles, making these mesoporous materials suitable for the rapid diffusion of molecules. This leads to superior separation applications over a wide range of molecular volumes [11].

Several attempts have been made to improve the performance of modified nanofilms to achieve a maximum permeation flux, solute rejection, and processing lifetime, which requires the determination of the optimal operating conditions for this process. Many parameters have been selected as the suitable conditions of operation, including; pH, feed solute concentration, feed temperature, content of embedded material, etc. [12]. Song et al. [13] researched the optimization of the spinning conditions on polyvinylidene fluoride (PVDF) fibers. They noted that the nonsolvent content, PVDF concentration, and diameter of the hollow fibers had a significant influence on the membrane distillation coefficient and its thermal efficiency. Khayet et al. [14] studied a strategy (Monte Carlo methods) to optimize the sweeping gas membrane distillation operation (SGMD).

To improve the performance of operations on the response, response surface methodology (RSM) can be employed, as it incorporates many statistical operations that are necessary for the response [15]. RSM has been used in the fields of engineering, biology, and other scientific disciplines. This method tests the effect of variables and interactions between them in a more robust and holistic manner compared to traditional methods that test the effect of one parameter at a time in a series of experimental studies [16]. Another advantage of RSM is the reduction in the experimental time, material consumption, and number of experiments required [17,18,19,20,21].

RSM based on the Box–Behnken design (BBD) was used to design runs and analyze the influences of operating variables for removing dyes (i.e., acid blue 25, disperse red 73, and methylene blue) using a composite nanofiltration membrane with an isoelectric point (IP) of 4.5 [22]. Also, RSM was successfully used with three variables (i.e., concentration, dye solution pH, and membrane composition) to improve the permeate flux and dye removal efficiency of a PES mixed-matrix nanofiltration membrane embedded with polymer-wrapped multi-walled carbon nanotubes (MWCNTs) [23]. Response surface methodology was used by Cretescu et al. [24] to improve the removal of Cu(II) from an aqueous solution and to determine the optimal conditions for sorption, such as the Cu(II) concentration, pH, and sorbent dose. Sufyan et al. [25] employed RSM based on the central composite design (CCD) to analyze the effects of operating variables such as feed flow rate, feed temperature, and permeate temperature using poly(vinylidene fluoride-co-hexafluoropropylene) (PVDF-HFP) flat sheet membranes for seawater desalination. The ANOVA results showed that the main and interaction factors had a significant impact on the permeate flux.

The Taguchi method is known for its strong performance in optimizing and designing high-quality processes using matrices. It is characterized by ease of use and wide application in engineering and scientific fields [26]. The Taguchi method is flexible and reproducible for analyzing experiments and determining the many simultaneous effects of parameters, with high efficiency compared to other statistical methods [27,28]. This procedure is distinctive in investigating the impacts of many parameters on a process and ranking the parameters based on their greater or lesser impacts [29,30]. Chen et al. [31] used this method coupled with an orthogonal fractional factorial method to improve the operating conditions for a continuous membrane distillation crystallization (CMDC) operation with a saturated brine feed solution. 

The Taguchi method was employed in the study of a systematic framework to test and optimize the performance of a sweep gas membrane distillation (SGMD) configuration in combination with a porous polyvinylidene fluoride-co-hexafluoropropylene (PVDF-co-HFP) membrane as reported by Nawras et al. [32]. Another study used response surface methodology (RSM) to find the optimal conditions for the removal of brilliant green dye using a combination of photocatalysis and ceramic nanofiltration processes, researching the effect of pH, catalyst loading, and time duration variables on the response [33].

This paper examines the optimization of operating parameters of the nanofiltration process with MCM-41 mesoporous material based on response surface methodology (RSM) and analysis of variance (ANOVA). The current work studied the optimization of various operating parameters (e.g., pH value [3,4,5,6,7,8,9,10,11], MCM-41 content [0–1%], and feed concentration [10–100 ppm]) in relation to the permeability of PES/MCM-41 membranes. Additionally, the interaction impacts between operating parameters were tested. The performance evaluation measured the influence of several operating variables (i.e., pH value, MCM-41 wt.% content, and feed concentration) for two dyes. Nanofiltration runs were performed to assess the permeability to provide practical applications for dye removal, as the experimental wastewater simulated the effluent that would arise from leather tanning and textile production.

## 2. Experimental Work 

### 2.1. Materials

The membrane materials were polyethersulfone (Radel^®^, with an average MW = 30,000 g mol^−1^) donated by Solvay Advanced Polymers (Brussels, Belgium). Dimethyl sulfoxide (DMSO, (CH_3_)_2_SO, MW = 78.13 g mol^−1^) was the polymer solvent, obtained from Sigma-Aldrich (St. Louis, MO, USA). Acid black 210 (AB-210, C_34_H_25_N_11_Na_2_O_11_S_3_, MW = 938.02 g mol^−1^) and rose bengal (RB, C_20_H_2_Cl_4_I_4_Na_2_O_5_, MW = 1017.64 g mol^−1^), were obtained from local markets (Baghdad, Iraq). 

### 2.2. Preparation of Composite Membranes

Seven blended membranes were fabricated via the classical noninduced phase separation technique (NIPS). The composition of the membranes is displayed in Table 1. Initially, the PES was dried at 60 °C for four hours to remove the moisture content and then dissolved with several contents ratios (0–1 wt.%) of MCM-41 in DMSO solvent at 40 °C and magnetically stirred overnight until a homogeneous casting solution was obtained. Following that, the proper amount was sprinkled on the glass plate and cast with a thickness of 200 µm. Thereafter, the prepared membrane was immersed in a deionized water bath at room temperature to finalize the phase inversion process. The fabricated membranes were repeatedly rinsed with deionized water and stored until further use. More details are presented in Table 1 [34].

### 2.3. Membranes Characteristics

The porosity of the samples was determined via gravimetric analysis, according to the following equation [35,36]:

(1) $$\varepsilon =\frac{W_{1}-W_{2}}{A\cdot T\cdot \rho }$$

where *ε* is the porosity (%); *W*_1_ and *W*_2_ are the weights of the wet and dry membranes (g), respectively; *A* is the effective membrane area (cm^2^); *T* is the membrane thickness (μm); and *ρ* is the water density (0.998 g/cm^3^ at 25 °C). Prior to measurements, the membrane samples of pristine and fabricated PES membranes were immersed in deionized water for 24 h at room temperature. After the water was wiped from the membranes, they were weighed. To estimate the average porosity of the membranes, triplicates of each sample were tested.

The static contact angle (CA) between the water drop and the membrane surface was measured at room temperature using a contact angle measuring device (CAM 110, Taiwan). The measurement started by placing a 4 µL water droplet on the surface of the flat membrane. The droplet image was captured automatically to represent the CA. To minimize the experimental errors, CAs for each sample were measured at three randomized locations, after which the mean value was presented.

**Scheme 1:**
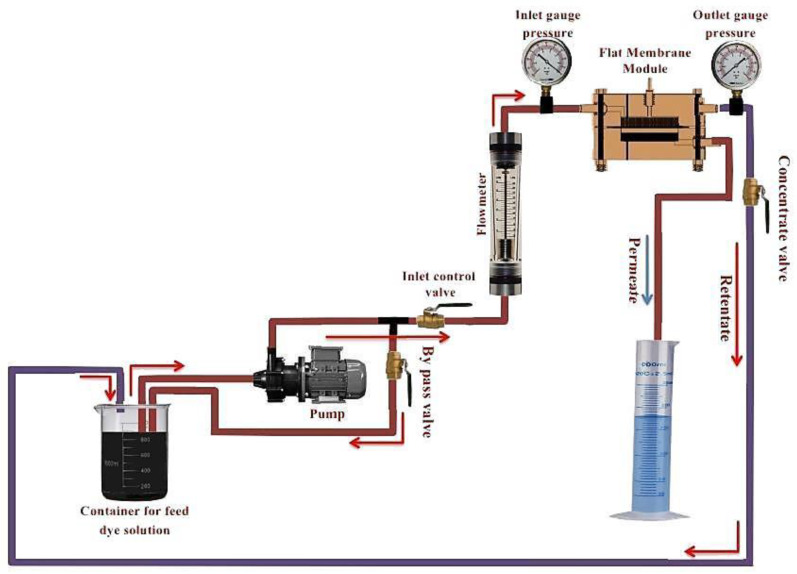
Schematic diagram of the cross-flow filtration system.

The permeation of the NF membranes was estimated under cross-flow filtration conditions. The apparatus setup was equipped with a membrane cell (7 × 7 × 4 cm^3^) having an effective membrane area of 16 cm^2^ (Scheme 1). First, each membrane sample was pressurized for 20 min at 4 bar to compact the membranes before the filtration tests. The pressure was then lowered to the operating pressure of 3 bars at 25 ± 1 °C, and the water permeability magnitudes were recorded. According to what was found in the literature for the concentration of dyes in wastewater [1,2], the separation tests were evaluated under four concentrations (i.e., 10, 50, 80, and 100 ppm) of aqueous solutions for each of the AB-210 and RB dyes. The pure water permeability was determined according to the following equation:

(2) $$J=\frac{V}{A\cdot t\cdot \Delta P}$$

where *J* is the pure water permeability of the membrane (L·m^−2^·hr^−1^·bar^−1^), *V* is the volume of the collected permeate (L), *t* is the time the permeate was collected (h), *A* is the membrane surface area (m^2^), and Δ*P* is the operational pressure (bar).

## 3. Results and Discussion

Variations in the membrane surface hydrophilicity were assessed by measuring the contact angle (CA) of the membranes. The contact angles of the raw PES membrane and PES with different amounts of MCM-41 (i.e., 0, 0.1 0.3, 0.5, 0.7, 0.8, and 1%) are shown in Table 1. The CA values of the top surface decreased from 69.2° for raw-PES to 40.5° as the amount of MCM-41 in the casting solution increased up to 0.5 wt.%. This indicates that MCM-41 (less than 0.5 wt.%) as an additive had a positive influence on the hydrophilicity of the fabricated membrane’s surface as the contact angle decreased. Compared to 69.2° for the pristine PES membrane, the contact angle of the modified membranes declined to 49.1°, 45.2°, and 40.5° for membranes of 0.1, 0.3, and 0.5 wt.%, respectively. Further increases in the loading weight of the additives caused a slight increase in the water contact angle values; however, they remained lower than those of the control PES. The 0.7, 0.8, and 1 wt.% modified membranes manifested contact angle values of approximately 52°, 58.5°, and 59.1°, respectively. This finding has been attributed to mesoporous aggregation MCM-41 and to the reduction of the effective area of mesoporous with high MCM-41 content. As a result, the number of MCM-41 functional groups on the membrane surface may be reduced. Lowering the contact angle to approximately 40.5° for the 0.5 wt.% membrane demonstrated that the mesoporous MCM-41 significantly enhanced the surface hydrophilicity characteristics of the membrane due to the hydrophilic property of fabricated membranes. Throughout the membrane formation process, MCM-41 moved to the membrane’s surface, causing it to retain the more hydrophilic property of the surface. This migration of MCM-41 gave rise to higher water adsorption, lower fouling, and enhanced permeability of the membrane. In addition, this influence of the MCM-41 content on the contact angle may result from varying the interface energy [37]. Table 2 illustrates the values of thickness, porosity, contact angle, and water permeability for the two membranes: (1) raw PES membrane and (2) PES with various amounts of MCM-41.

## 4. Optimization and Modeling Process

Employing membranes often creates energy efficiency problems. Therefore, the primary goal of the optimization and modeling process is to reduce the energy expended while maintaining high permeance during the operation. The purpose of this work is the optimization of the operating conditions to use PES/MCM-41 flat sheet membranes for NF process applications. This research studied the impact of the operational variables, such as the feed pH values (3–11), added MCM-41 content (0–1 wt.%) in the casting solution, and feed dye concentration (10–100 ppm) for each of the studied dyes, AB-210 and RB, as well as their interactions on the PES membrane permeability. Determining the optimum operating variables will allow for the most effective NF process to produce a suitable value of permeability.

### 4.1. Optimization of Operating Parameters 

A number of experiments were performed using the NF membrane process by adjusting one parameter at a time to identify the required set of operating variables while holding the other variables constant. The significance of optimizing operational parameters was to determine how independent parameters, such as pH value, AB-210 or RB dye concentration, and MCM-41weight ratio content influenced the permeate system flow.

The analyses were performed using the software MINITAB^®^17. Due to their better membrane performance, the modified PES/MCM-41 flat sheets utilized in this process had a 58.6° contact angle under 3 bar pressure. The developed optimization design for membrane permeability response was verified statistically via the analysis of variance (ANOVA) technique, which presented good coefficient of determination values, *R*^2^ = 0.8828 and 0.8868, for AB-210 and RB dyes, respectively. Table 3 illustrates the experimental codes of various parameters, indicating either low or high levels after their units. To avoid any methodological error in the optimization design, a random distribution was used, which facilitated obtaining optimal values of the operating variables for the NF process.

### 4.2. ANOVA Models

An ANOVA represents a number of statistical models utilized to analyze the variations between several parameters; more precisely, it is used to define the impact that independent parameters have on the dependent variables in a regression analysis. The application of an ANOVA can determine the optimum set of process operating parameters more rigorously by examining the relative significance among the variables. ANOVA calculations were handled using the software package MINITAB^®^17, with a level of significance of 5% needed to consider a variable necessary to the operational process. Table 4 indicates the ANOVA results for the permeability as a function of the MCM-41 weight ratios, dye concentrations, and feed pH values. In the ANOVA table, there is a *p*-value for each independent variable in the design. When the *p*-value is less than 5%, the variables are statistically significant [38].

The regression equation (Equation (3)) for AB-210 shows the influence of the significant variables on the permeability (Y) during the NF process in terms of coded levels:

(3) $$Y=45.28-3.7X_{4}-8.29X_{2}+24.45X_{1}+1.12{\left(X_{4}\right)}^{2}-0.48{\left(X_{2}\right)}^{2}-21.42{\left(X_{1}\right)}^{2}-2.67X_{4}X_{2}-3.33X_{2}X_{1}$$

The regression equation for the RB dye (Equation (4)) is as follows:

(4) $$Y=42.06-4.65X_{4}-8.84X_{3}+24.02X_{1}+1.4{\left(X_{4}\right)}^{2}-0.38{\left(X_{3}\right)}^{2}-19.68{\left(X_{1}\right)}^{2}-3.21X_{4}X_{3}-3.76X_{3}X_{1}\;\;\;\;\;\;\;\;\;\;\;\;\;\;\;\;$$

The ANOVA for the membrane permeability and response surface regression for the AB-210 and RB dyes is displayed in Table 5 and Table 6, respectively: 

### 4.3. Evaluating and Analyzing Results 

#### 4.3.1. Effect Plot

##### 4.3.1.1. Pareto Chart

**Figure 1:**
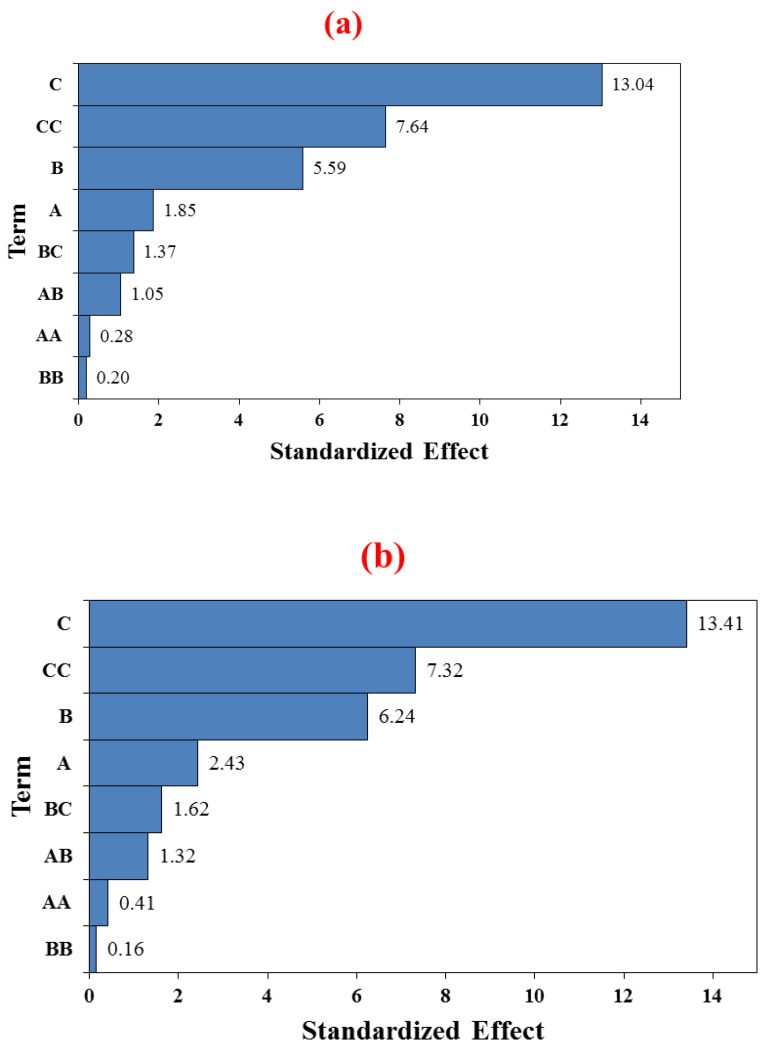
Pareto chart of the standardized effects of three parameters—pH (A), concentration (B), and MCM-41 wt.% (C)—on the permeability of (**a**) AB-210, and (**b**) RB dyes.

Figure 1 represents the Pareto chart of the standardized effects for the permeability of both dyes, which relates to the effect on the permeability of the main factors, including the feed solution pH (factor A), feed concentration of dyes (factor B), added MCM-41 weight ratios into the dope solution (factor C), and some interaction factors. Consequently, the standardized effect value for each coefficient must be more than the critical value, 2.02, to be statistically significant at the 5% level. The importance of the MCM-41 wt.% content is evident through the term (CC), which represents the quadratic effect. The largest standardized effect is the MCM-41 wt.% content (factor C).

The content of the MCM-41 wt.% in the casting solution (factor C) and the concentration of the AB-210 dye solution (factor B) both exceeded the critical value (i.e., they were significant). However, the estimated main effect of the feed pH (factor A) was small and not significant, as can be seen in Figure 1a. In the case of the RB dye solution (Figure 1b), the main effects MCM-41 wt.% content (factor C), dye concentration (factor B), and pH solution (factor A) cross the reference line of 2.02, indicating that they were essential to this process.

##### 4.3.1.2. Main Effects Plot

**Figure 2:**
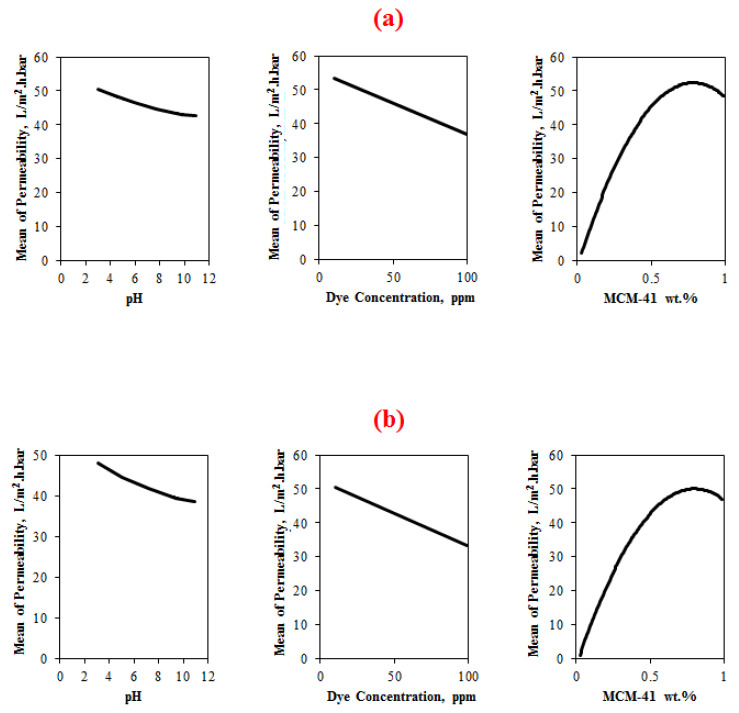
Main effect plot of the pH, concentration, and MCM-41 wt.% on the permeability of (**a**) AB—210, and (**b**) RB dyes.

Figure 2 shows the main effects plot for the dyes’ permeability. The main effects plot indicates that the MCM-41 wt.% content was the most influential factor, followed by the concentration of the dyes and the pH level. As the MCM-41 wt.% content increased to a moderate level of approximately 0.8 wt.%, the permeability rose and then decreased at 1 wt.% of MCM-41. As can be seen from Figure 2, the permeation increased when the feed concentration varied from high to low (i.e., from 100 to 10 ppm).

Figure 2a shows that the pH of the AB-210 dye solution had almost no influence on the permeability and that the pH level could be any value from 3–11. The pH of the RB dye solution had very little influence on the permeability, and the value of the pH at maximum permeability was 3, as shown in Figure 2b.

##### 4.3.1.3. Interaction Plot

**Figure 3:**
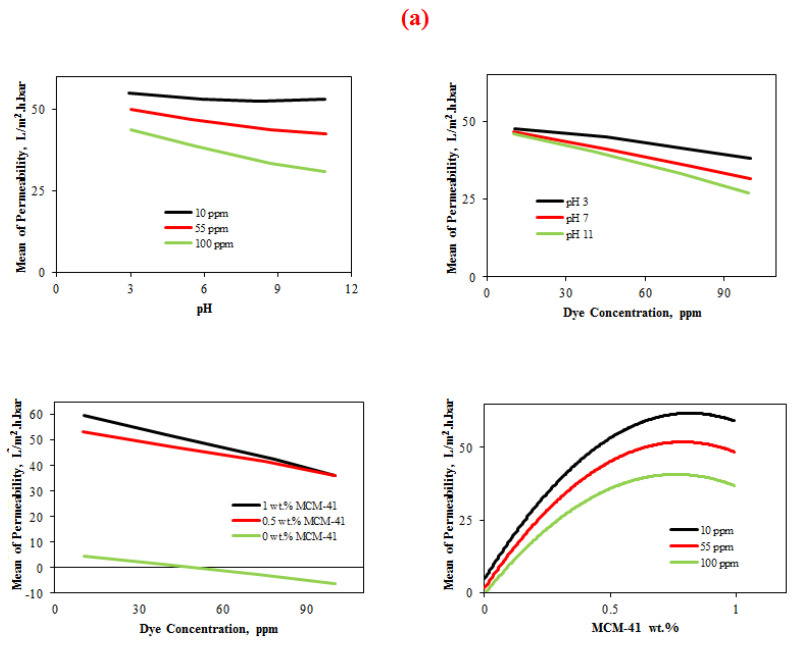

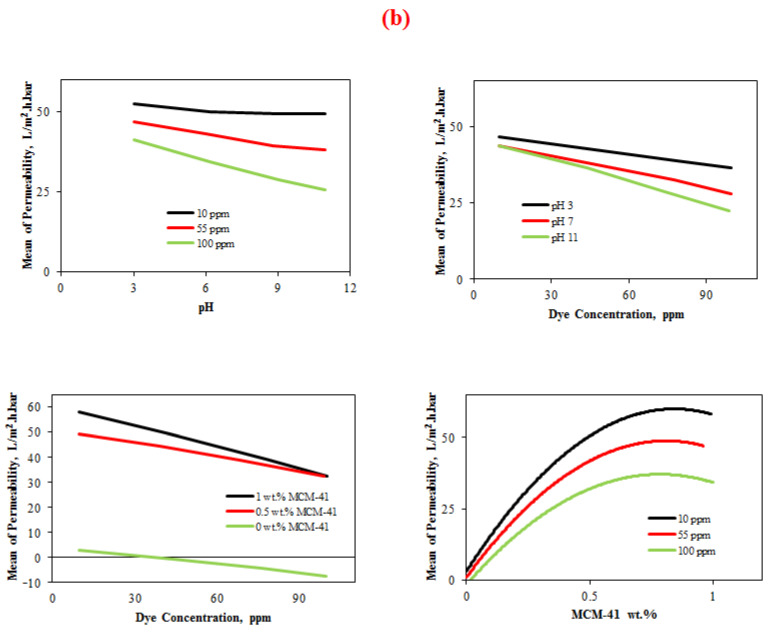
Interaction plots of the pH, concentration, and MCM-41 wt.% on the permeability of (**a**) AB—210, (**b**) RB dyes.

Figure 3 shows the interaction graph for all three process variables. Some interaction was found between the variables. The interaction plot illustrates that between the MCM-41 weight ratio and dye concentration, the maximum permeability was observed for an MCM-41 wt.% of approximately 0.8 wt.% in the casting solution at 10 ppm, and the minimum permeability was observed for a zero weight ratio of MCM-41 (pure PES) at a dye concentration of 100 ppm. The interaction plot between the dye concentration and MCM-41 weight ratio for permeability showed that as the concentration decreased, the permeability increased for all MCM-41 weight ratios.

Meanwhile, the interaction plot between the feed concentration and the pH demonstrated that the permeability of all the pH solutions significantly increased with a decrease in the feed concentration. Maximum permeability was observed for all feed pH levels at a dye concentration of 10 ppm. It is clear from the graph that the interactions for the AB-210 dye were almost identical to the RB dye, with little difference in permeability.

#### 4.3.2. Residual Plot

##### 4.3.2.1. Normal Probability Plot of Residuals

**Figure 4:**
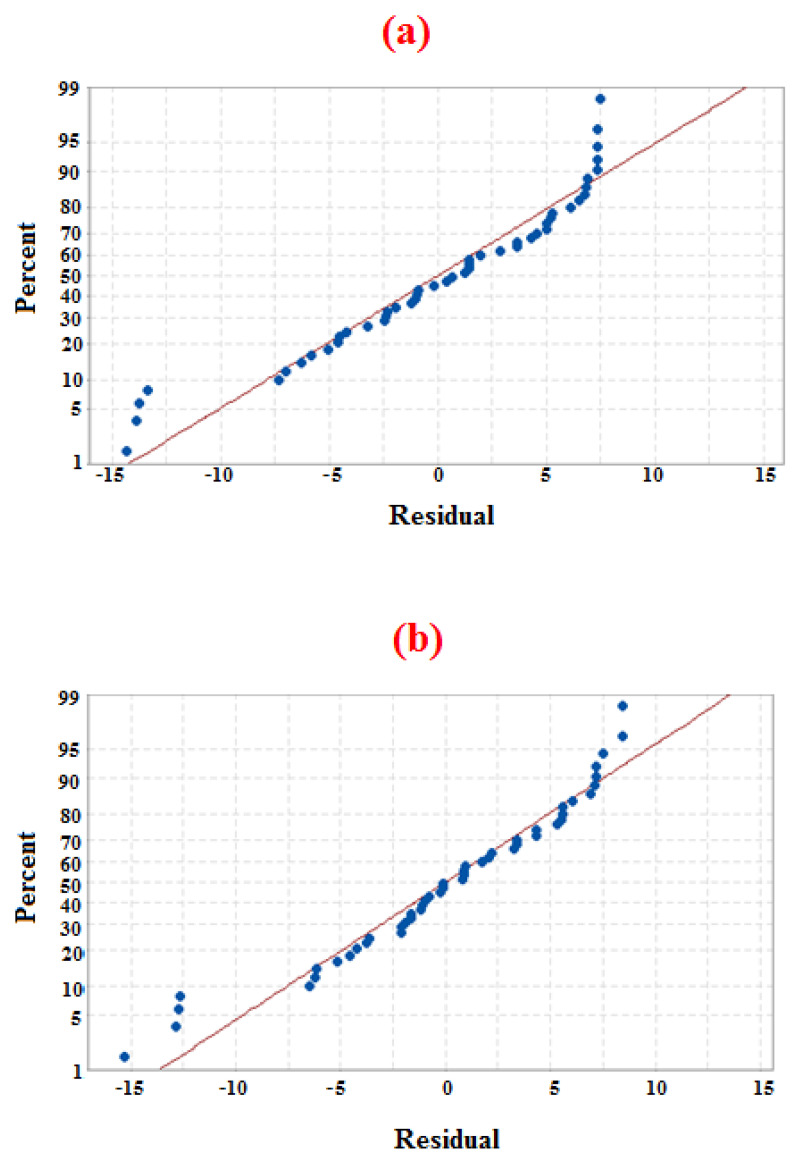
Normal probability plot of the residuals for the permeability of (**a**) AB—210, and (**b**) RB dyes.

The normal probability graph is a diagrammatic tool for determining whether or not a data set is naturally diffused. Data is sketched in a mode where the points can fall along a straight line versus in a theoretical normal distribution. Points that are further from the straight line can be ignored because those values are not valid. In the plotted results, all data residuals are typically spread so that an approximately straight line is created by the total points, as can be seen in Figure 4.

##### 4.3.2.2. Residuals Versus Order and Fits Plot

**Figure 5:**
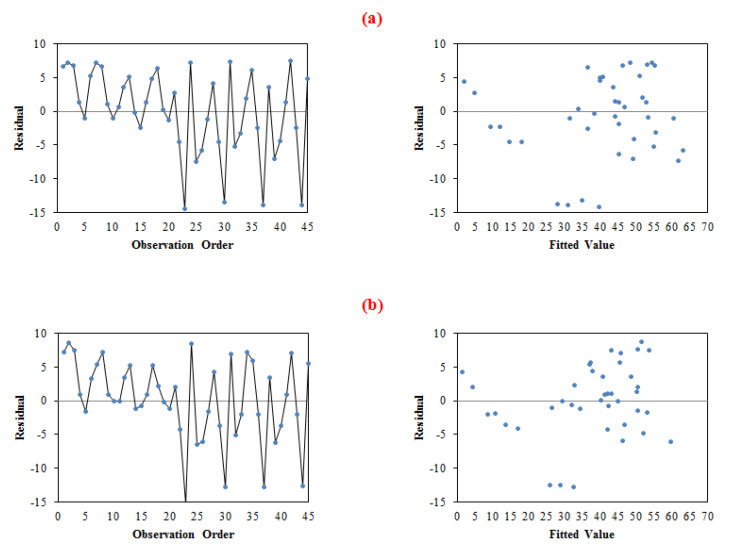
Residuals versus the observation orders and fitted values plot for the permeability of (**a**) AB-210, and (**b**) RB dyes.

A residual graph is used to measure the fitness of ANOVA values. Figure 5 shows the residuals versus the independent parameters. Key assumptions about errors include that they are independent of each other and that the simplest way to produce an independent error is to plan the randomization of experimental trials. Additionally, the error discrepancy does not change the varying degrees of the parameters nor does it change based on the predicted response values.

#### 4.3.3. Response Surface Analysis

**Figure 6:**
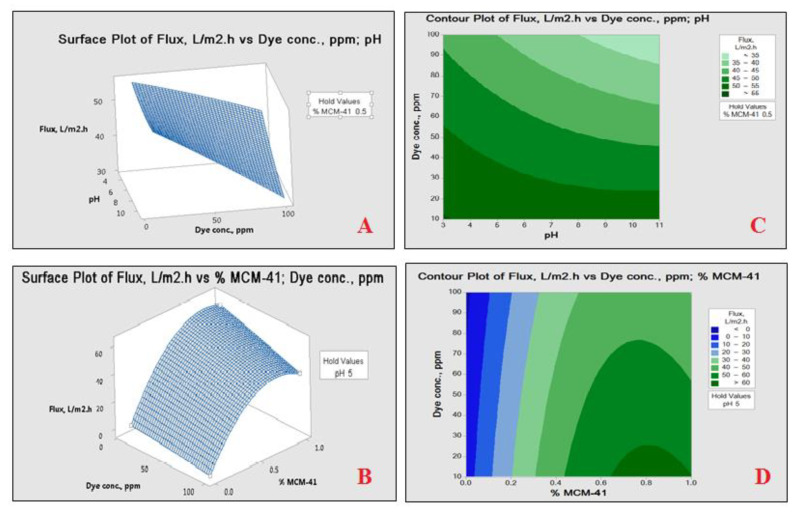
Three-dimensional response surface plot (**A**,**B**), and two-dimensional response contour plot (**C**,**D**) of the permeability as a function of the MCM-41 weight ratio, feed pH, and dye concentration for AB-210 dye.

**Figure 7:**
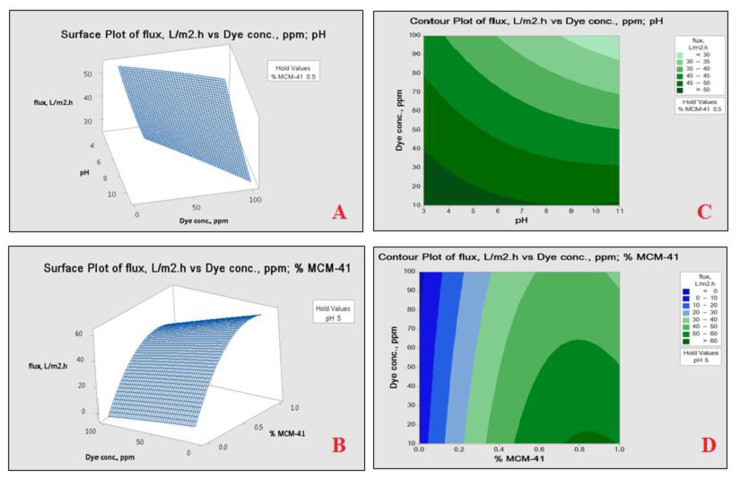
Three-dimensional response surface plot (**A**,**B**), and two-dimensional response contour plot (**C**,**D**) of the permeability as a function of the MCM-41 weight ratio, feed pH, and dye concentration for RB dye.

The response of the permeability of dyes can be described as a solid in three-dimensional surface space, where the permeability is plotted versus the limits of two variables. The main point of the response surface graph is to identify the optimum operating conditions represented by the pH value of the feed, MCM-41 weight ratios, and dye concentration (either AB-210 or RB) that result in the maximum permeability. Three-dimensional response surface plots and two-dimensional response contour plots were established using the fitted model, as shown in Figure 6 and Figure 7.

The response surface plot and contour plot (Figure 6A,C) of the AB-210 dye permeability response function revealed that decreasing the feed pH and decreasing the feed concentration resulted in a higher permeability at a constant MCM-41 weight ratio (i.e., 0.5 wt.%). However, as expected, the effect on the permeation of varying the pH value was not significant for lower levels of concentration. Figure 6B,D, presented the effect of two factors, MCM-41 weight ratio, and concentration when the third factor (pH) was held constant at 5. The rise in the MCM-41 wt.% to 0.8 wt.% increased the permeability to more than 60 LMH/bar, while varying the concentration had little influence on the permeability. For higher ratios of MSM-41 content (MCM-41 wt.% > 0.8 wt.%), the trend was reversed. Also, the response of the permeability demonstrated that the interaction effect between the MCM-41 weight ratio and concentration decreased. This agrees with the observations for both dyes previously mentioned in the “Interaction plot,” Section 4.3.1.3.

The response surface analysis for RB dye was nearly similar to that of AB-210 dye. Figure 7A,C, presented the effect of the two factors, feed pH and feed concentrations, when the third-factor MCM-41 weight ratio was held at 0.5 wt.%. As it can be seen in the figure, the lower pH shows some minor effects on response with RB dye for lower levels of concentration (See “Interaction plot,” Section 4.3.1.3). Also, in Figure 7B,D, the effect of MCM-41 weight ratio and concentration is depicted at constant pH 5. It is noted that increasing MCM-41 wt.% resulted in the higher permeability. The highest values of permeability were obtained in the 0.8 wt.% MCM-41.

### 4.4. Optimization of the NF Membrane Permeability

**Figure 8:**
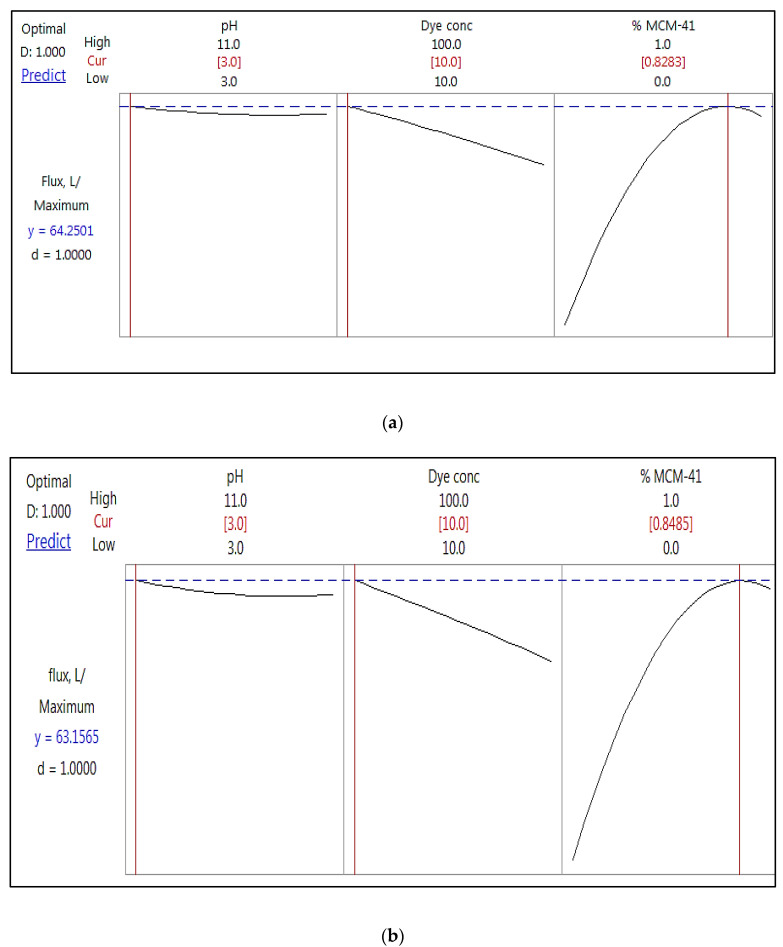
Optimization plot of the process parameters for the maximum permeability for (**a**) AB-210, and (**b**) RB dyes.

Figure 8 illustrates the optimization design of the membrane permeability with various levels of the three parameters (i.e., MCM-41 wt.% content, dye concentration, and feed pH values). The optimization chart was created using the collected data from the MINITAB^®^17 computer software. The optimization results are shown in the left column, while the optimum value for each variable is illustrated in the center of the top row (in red).

Figure 8a predicts that for the AB-210 dye solution, the optimum operating conditions are a feed pH value of 3, dye concentration of 10 ppm, and 0.828 wt.% MCM-41 content. The maximum permeability is estimated to be 64.25 L.m^−2^.h^−1^. It can be concluded that the primary factor in this process is that MCM-41 weight ratios had been added to the casting solution, followed by the secondary factor, dye concentrations. As indicated by the presence of a horizontal line, the feed pH values had no significant influence on the permeability.

As illustrated in Figure 8b, the optimal combination of parameters to maximize the RB dye permeability was a feed pH value of 3, feed concentration of 10 ppm, and MCM wt.% of 0.849 wt.%. The highest values of permeability were obtained at 63.16 L.m^−2^.h^−1^. As with the AB-210 dye, the principal factor in this process was the added MCM-41 weight ratios in the casting solution, followed by the dye concentrations as the secondary factor. When a line has a small deflection from the horizontal, as does the line for the pH, it can affect the response. The pH value had a lesser impact on the permeability than the other factors as signified by the less steep slope of the line.

The permeation of dye solutions rises when using manufactured membranes with various concentrations of mesoporous MCM-41 at different solution concentrations. The higher permeate of the manufactured membranes after the addition of MCM-41 weight ratios might result from the increase in the hydrophilicity and morphological traits of the manufactured membranes (see Table 2). Membranes that have the highest permeability are the ones with higher hydrophilicity and porosity. A relative decrease in the permeability was observed when increasing the initial feed concentration for both the AB-210 and RB dyes. This decrease has been described in the literature related to the adsorption of dye molecules, where a cake layer forms on the surface of the membrane, preventing the passage of water through the membrane and reducing the flow [39].

On the other hand, there was little effect caused by the acidity of the two dyes, with no significant influence on the permeability of the AB-210 dye. However, with the RB dye, the acid had a minor positive effect on the permeability.

## 5. Conclusions

MCM-41 mesoporous material was employed to modify flat sheet PES nanofiltration (NF) membranes for dye removal. Various loading ratios (i.e., 0, 0.1, 0.3, 0.5, 0.7, 0.8, and 1 wt.%) of MCM-41 were impregnated within the PES polymeric matrix via the classical non-solvent induced phase separation (NIPS) technique. All control and composite membranes were comprehensively described in terms of the permeation and separation characteristics using two dyes models, AB-210 and RB.

Optimization techniques were utilized to determine the operating parameters that most affect the permeability of PES/MCM-41 wt.% flat sheet membranes in the nanofiltration process. The three parameters identified as having the most influence on the permeance were the MCM-41 content (%), dye concentration (either AB-210 or RB), and pH values. The experimental design allowed for studying the effect of three levels for each of the two dyes. These experiments found that the MCM-41 content and dye concentration were the most significant parameters to influence the permeability. In contrast, the feed pH of the AB-210 dye had no significant effect on the permeability, but the pH slightly influenced the permeability when using the RB dye.

The design also allowed for specifying the optimal values of the three factors based on several experiments. The optimum parameters were MCM-41 content (nearly 0.8 wt.%) and feed dye concentration (10 ppm) for the studied dyes, and a pH value (3) for the RB dye only; for the AB-210 dye, the pH value could range from 3–11. Under these conditions, a maximum permeability of 64.25 and 63.16 L.m^-2^.h^-1^.bar^-1^ for the AB-210 and RB dyes, respectively, was achieved.

## Figures and Tables

**Table 1:** Compositions of PES/MCM-41 casting solutions.

Membrane Code	Casting Solution Compositions (wt.%)		Solvents (DMSO) (wt.%)
	PES	MCM-41	
M0	21	0	79
M1	21	0.1	79.9
M2	21	0.3	79.7
M3	21	0.5	79.5
M4	21	0.7	79.3
M5	21	0.8	79.2
M6	21	1	78

**Table 2:** Effect of MCM-41 wt.% on the porosity, water contact angle, and water permeability of PES membranes.

Membrane Code	Thickness * (µm)	Porosity (%)	Average Contact Angle (°)	Pure WATER Permeability (LMH/bar)
M0	151.12 ± 2.46	26.06 ± 1.25	69.2 ± 3.80	10
M1	143.69 ± 1.19	66.90 ± 0.63	49.1 ± 2.09	15
M2	128.61 ± 2.35	68.20 ± 1.00	45.2 ± 2.50	38.37
M3	119.83 ± 1.06	82.80 ± 0.51	40.5 ± 0.50	77.8
M4	95.55 ± 1.22	73.20 ± 0.38	52.0 ± 1.60	65.2
M5	91.20 ± 0.79	75.76 ± 0.47	58.6 ± 0.97	66.5
M6	87.99 ± 1.11	76.60 ± 0.91	59.1 ± 1.01	70

* The thickness of the membranes was measured by using an SEM device.

**Table 3:** Values and codes of the experimental variables at various levels.

Parameters	Code	Units	Low level	High Level
MCM-41 content	X1	wt.%	0	1
AB 210 dye concentration	X2	ppm	10	100
RB dye concentration	X3	ppm	10	100
pH of dye solution	X4	-	3	11
permeability	Y	LMH/bar	Target	

**Table 4:** Analysis of variance (ANOVA) for the permeability of (a) AB-210, and (b) RB dyes.

**Source**				**DF**	**Adj SS**	**Adj MS**	**F-Value**	***p*-Value**
Model				8	13,166.7	1645.84	36.70	0.000
Linear				3	9347.7	3115.91	69.49	0.000
pH				1	155.3	155.32	3.46	0.070
Concentration				1	1417.1	1417.11	31.60	0.000
% MCM-41				1	7680.8	7680.85	171.29	0.000
Square				3	2825.2	941.74	21.00	0.000
pH × pH				1	4.9	4.86	0.11	0.744
Dye conc., ppm × Dye conc.,ppm				1	2.0	2.03	0.05	0.833
% MCM-41 × % MCM-41				1	2638.6	2638.55	58.84	0.000
2-Way Interaction				2	137.6	68.78	1.53	0.228
pH × conc.				1	52.3	52.34	1.17	0.287
Conc. × % MCM-41				1	83.5	83.51	1.86	0.180
Error				39	1748.8	44.84		
Lack-of-Fit				35	1748.8	49.96		
Pure Error				4	0.0	0.00		
Total				47	14,915.5			
S	R-sq	R-sq(adj)	R-sq(pred)					**(a) AB 210 dye**
6.69627	88.28%	85.87%	83.95%					
**Source**				**DF**	**Adj SS**	**Adj MS**	**F-Value**	***p*-Value**
Model				8	12,533.6	1566.70	38.17	0.000
Linear				3	9390.0	3130.01	76.26	0.000
pH				1	245.9	245.90	5.99	0.019
Concentration				1	1610.5	1610.54	39.24	0.000
% MCM-41				1	7408.6	7408.56	180.51	0.000
Square				3	2402.6	800.88	19.51	0.000
pH × pH				1	7.6	7.57	0.18	0.670
Dye conc., ppm × Dye conc.,ppm				1	1.3	1.28	0.03	0.861
% MCM-41 × % MCM-41				1	2226.4	2226.41	54.25	0.000
2-Way Interaction				2	184.1	92.06	2.24	0.120
pH × conc.				1	75.6	75.57	1.84	0.183
conc. × % MCM-41				1	106.2	106.24	2.59	0.116
Error				39	1600.7	41.04		
Lack-of-Fit				35	1599.7	45.71	186.55	0.000
Pure Error				4	1.0	0.24		
Total				47	14,134.2			
S	R-sq	R-sq(adj)	R-sq(pred)					**(b) RB dye**
6.40645	88.68%	86.35%	84.43%					

DF: degrees of freedom; Adj SS: adjusted sum of squares; Adj MS: adjusted sum of mean squares; F: measure of variability within the sample; P: percentage of contribution.

**Table 5:** ANOVA for the membrane permeability of (a) AB-210, and (b) RB dyes.

**(a) AB 210 Dye**									
**Coded Coefficients**									
**Term**		**Coef**	**SE Coef**		**T-Value**		**p-Value**		**VIF**
Constant		45.28	2.32		19.49		0.000		
pH		−3.70	1.99		−1.86		0.070		1.14
Concentration		−8.29	1.47		−5.62		0.000		1.32
% MCM-41		24.45	1.87		13.09		0.000		1.03
pH × pH		1.12	3.40		0.33		0.744		1.09
Conc. × Conc.		−0.48	2.24		−0.21		0.833		1.00
% MCM-41 × % MCM-41		−21.42	2.79		−7.67		0.000		1.16
pH × Conc.		−2.67	2.47		−1.08		0.287		1.34
Conc. × % MCM-41		−3.33	2.44		−1.36		0.180		1.02
**Fits and Diagnostics for Unusual Observations**									
**Obs**	**permeability**	**Fit**		**Resid**		**Std Resid**			
23	25.00	39.35		−14.35		−2.31		R	
30	21.40	34.82		−13.42		−2.10		R	
37	17.00	30.94		−13.94		−2.15		R	
44	14.30	28.11		−13.81		−2.19		R	
**(b) RB Dye**									
**Term**		**Coef**	**SE Coef**		**T-Value**		**p-Value**		**VIF**
Constant		42.06	2.22		18.92		0.000		
pH		−4.65	1.90		−2.45		0.019		1.14
Concentration		−8.84	1.41		−6.26		0.000		1.32
% MCM-41		24.02	1.79		13.44		0.000		1.03
pH × pH		1.40	3.25		0.43		0.670		1.09
Conc. × Conc.		−0.38	2.14		−0.18		0.861		1.00
% MCM-41 × % MCM-41		−19.68	2.67		−7.37		0.000		1.16
pH × Conc.		−3.21	2.37		−1.36		0.183		1.34
Conc. × % MCM-41		−3.76	2.33		−1.61		0.116		1.02
**Fits and Diagnostics for Unusual Observations**									
**Obs**	**permeability**	**Fit**		**Resid**		**Std Resid**			
23	22.00	37.33		−15.33		−2.58		R	
30	19.70	32.61		−12.91		−2.11		R	
37	16.00	28.68		−12.68		−2.05		R	
44	13.10	25.87		−12.77		−2.12		R	

**Table 6:** Predicted response for the membrane permeability of (a) AB-210, and (b) RB dyes.

**Response Optimization: permeability (L.m^−2^.hr^−1^.bar^−1^)**										**(a) AB 210 dye**									
**Parameters**																			
**Response**	**Goal**				**Lower**				**Target**		**Upper**				**Weight**				**Importance**
permeability	Maximum				4.9				62						1				1
**Solution**																			
**Solution**		**pH**				**Concentration**				**% MCM-41**				permeability **Fit**				**Composite Desirability**	
1		3				10				0.828				64.2501				1	
**Multiple Response Prediction**																			
**Variable**										**Setting**									
pH										3									
Concentration										10									
% MCM-41										0.828									
**Response**			**Fit**				**SE Fit**					**95% CI**				**95% PI**			
permeability			64.25				4.52					(55.11; 73.39)				(47.91; 80.59)			
**Response Optimization: permeability (L.m^−2^.hr^−1^.bar^−1^)**										**(b) RB dye**									
**Parameters**																			
**Response**	**Goal**				**Lower**				**Target**		**Upper**				Weight				Importance
permeability	Maximum				4.3				60.5						1				1
Solution																			
**Solution**		**pH**				**Conc.**				**% MCM-41**				permeability **Fit**				**Composite Desirability**	
1		3				10				0.848				63.16				1	
**Multiple Response Prediction**																			
**Variable**										**Setting**									
pH										3									
Concentration										10									
% MCM-41										0.848									
**Response**				**Fit**				**SE Fit**					**95% CI**				**95% PI**		
permeability				63.16				4.41					(54.23; 72.08)				(47.42; 78.89)		

## Data Availability

The data supporting reported results can be found in the PhD thesis of Rana J. Khadhim, University of Technology, Baghdad, Iraq.
